# Total and Free Sugar Content of Pre-Packaged Foods and Non-Alcoholic Beverages in Slovenia

**DOI:** 10.3390/nu10020151

**Published:** 2018-01-30

**Authors:** Nina Zupanič, Krista Miklavec, Anita Kušar, Katja Žmitek, Nataša Fidler Mis, Igor Pravst

**Affiliations:** 1 Nutrition Institute, Tržaška cesta 40, Ljubljana SI-1000, Slovenia; nina.zupanic@nutris.org (N.Z.); krista.miklavec@nutris.org (K.M.); anita.kusar@nutris.org (A.K.); katja.zmitek@vist.si (K.Z.); 2 VIST—Higher School of Applied Sciences, Gerbičeva cesta 51a, Ljubljana SI-1000, Slovenia; 3 Department of Gastroenterology, Hepatology and Nutrition, University Children’s Hospital, University Medical Centre Ljubljana, Ljubljana 1000, Slovenia; natasa.fidler@kclj.si

**Keywords:** free sugar, sugars, food composition, sales data, nutrition label, Slovenia

## Abstract

Scientific evidence of the association between free sugar consumption and several adverse health effects has led many public health institutions to take measures to limit the intake of added or free sugar. Monitoring the efficiency of such policies and the amount of free sugar consumed requires precise knowledge of free sugar content in different food products. To meet this need, our cross-sectional study aimed at assessing free sugar content for 10,674 pre-packaged food items available from major Slovenian food stores during data collection in 2015. Together, 52.6% of all analyzed products contained free sugar, which accounted for an average of 57.5% of the total sugar content. Food categories with the highest median free sugar content were: honey and syrups (78.0 g/100 g), jellies (62.9 g/100 g), chocolate and sweets (44.6 g/100 g), jam and spreads (35.9 g/100 g), and cereal bars (23.8 g/100 g). Using year-round sales data provided by the retailers, the data showed that chocolate, sweets, and soft drinks alone accounted for more than 50% of all free sugar sold on the Slovenian market. The results of this study can be used to prepare more targeted interventions and efficient dietary recommendations.

## 1. Introduction

A worldwide pandemic of obesity and metabolic diseases has raised concerns about the adverse effects of sugar intake above the recommended levels. There is epidemiological evidence available that links the consumption of added and/or free sugar and especially sugar-sweetened beverages (SSBs) with several undesirable health effects such as dental caries [1,2,3,4,5], insulin resistance [6], type 2 diabetes [7,8], metabolic syndrome [7,9,10], cardio-vascular disease [11,12,13,14], fatty liver [15,16,17], and dyslipidaemia [14,18]. Additionally, several clinical dietary intervention studies have indicated that diets with commonly-consumed levels of added sugars impair glucose and lipid metabolism, and increase several other risk factors associated with metabolic diseases [19,20]. However, some controversy remains as to whether these unfavorable health outcomes are a direct consequence of excessive consumption of free sugar, or rather a result of excessive energy intake [21,22].

Several governmental and public health organizations have issued recommendations to reduce the intake of free sugars. The new WHO (World Health Organization) guidelines on sugar intake published in 2015 recommended a reduction in free sugar intake for children and adults to less than 10% of total energy intake (strong recommendation), while a further reduction of the intake of free sugar to a maximum of 5% of total energy intake was recommended conditionally for additional health benefits [23]. In accordance with a recent European Society for Paediatric Gastroenterology, Hepatology and Nutrition (ESPGHAN) position paper, free sugar intake should be reduced to <5% energy intake in children and adolescents aged ≥2–18 years, and should be even lower in infants and toddlers <2 years [24]. Similarly, The UK Scientific Advisory Committee on Nutrition (SACN) recommends a free sugar intake lower than 5% of total energy from two years old into adulthood [25], while the American Heart Association (AHA) recommends children consume ≤25 g (100 kcal) of added sugar per day and to avoid added sugar completely in children <2 years of age [26].

Free sugars are the prime target of potential nutrition interventions since they provide energy with little or no nutritional value. The term free sugar in this paper follows the updated WHO definition and refers to all monosaccharaides and disaccharides added to foods and beverages by the manufacturer, cook, or consumer, and sugars naturally present in honey, syrups, fruit juices, and fruit concentrates [23]. This definition does not include naturally occurring or intrinsic sugars, which are stored within the cells of intact fruits, vegetables, or involve lactose in milk or unsweetened dairy products.

The review of European-based nation-wide representative studies on added and total sugar intake showed that sugars represent a significant proportion of the total daily energy intake. The reported consumption of added sugars was especially high among children, ranging from 11.0% to almost 16.8% of daily energy intake, while in adults it ranged between 7.3–11.4% [27]. However, in recent years, the attitude of some consumers towards free/added sugar has changed. An increasing number of consumer groups are initiating a reduction of free sugar intake in their diets [28]; however, doing so might prove difficult due to the lack of accessible information on the amount of added or free sugar in foods and beverages on the market [29]. Major progress in the EU (European Union) has been made with the new requirements for mandatory nutrition labelling of pre-packaged foods, which must provide the information about the total sugar content, as instructed by the Regulation 1169/2011 in the Provision of Food Information to Consumers [30]. This rule was enacted in December 2016; however, prior to the rule, nutrition declarations had been voluntary, except on foods carrying nutrition or health claims. This regulatory change has been particularly important for member states with the lowest penetration of nutrition declaration on food labels such as Slovenia [31,32]. While the nutrition declaration now provides information on (total) sugar content, the EU regulation does not require labelling of added or free sugar content. One of the major challenges encountered with potential free sugar labelling is its chemical indistinguishability from naturally-occurring sugar; therefore, there is no simple and accurate analytical method available that would discriminate between naturally-occurring sugar and added or free sugar. Nevertheless, the US Food and Drug Administration (FDA) has updated the nutrition facts label requirements where all nutritional labels on food packages will have to provide information on the amount of added sugar by 2019 [33].

In Slovenia, high sugar consumption and obesity prevalence are particularly concerning. According to the last available data, the mean intake of free sugar in Slovenian adolescents (aged 15–16) was 130 g/day in boys and 110 g/day in girls (16% and 17% of the daily energy intake, respectively) [34,35]. Results of a cross-national HBSC study (Health Behavior in School-aged Children), published by WHO in 2017, showed a high prevalence of obesity among Slovenian adolescents aged 11, 13, and 15 years. In 2014, 3.1% of girls and 7.8% of boys were obese, ranking Slovenia 4th among the 31 European countries included in the survey. The data were especially worrisome for 11 years old boys, where the prevalence of obesity was as high as 10.4% [36]. To tackle the problem of obesity on a national level, the Slovenian Ministry of Health has issued a strategic plan to gradually diminish the percentage of overweight and obese individuals. One of the main goals of The Slovenian Resolution on Nutrition and Physical Activity for Health (SRNPAH) 2015–2025 is to reduce the intake of free sugars by 15%, with an emphasis on soft drinks, sweets, and desserts [37].

To enable more accurate studies in monitoring the intake of total and free sugar in the Slovenian population, this study aimed to establish a comprehensive database of estimated free sugar content in the Slovenian food supply, focusing on pre-packaged foods. Using a step-by-step approach adapted from Bernstein and colleagues [38], free sugar contents were calculated by combining the data from the nutrition declarations and ingredient lists of foods and beverages in the food supply. Such data, currently unavailable, would serve as a reference point for future studies, as well as for monitoring the efficiency of dietary recommendations and public health interventions.

## 2. Material and Methods

### 2.1. Data Collection

As a part of our previously conducted study described in Reference [39], nutritional composition data were collected between January and February 2015 from the supermarket stores of the three major grocery chains with the largest nation-wide network of shops (Spar, Mercator, and Hofer). Sampling was performed in Ljubljana, Slovenia. In agreement with the retailers, all pre-packaged products with a unique European/International Article Number (EAN) barcode were systematically photographed and recorded in an online Composition and Labelling Information System (CLAS) database [40]. Such database was first compiled in 2011 and has been described elsewhere in References [32,41]. The CLAS Content Management System is supported by a specially developed mobile application, which enables the photographing of foods, data transfer to the CLAS database, and digital recognition of EAN codes to speed up the database formation and to avoid duplicate entries. During data collection in 2015, information on a total of 10,674 unique items were collected, including the product name, company, brand, list of ingredients, nutritional values, packaging volume, price, and EAN barcode. Flour, spices, sugar, food supplements, as well as all alcoholic beverages were excluded from the data acquisition.

All data entered into the CLAS database was rechecked and each product assigned to one of the 49 predefined food categories using a classification system developed by Dunford and colleagues [42], which was developed as part of the Global Food Monitoring Initiative. The categories used in this paper were as follows: baby foods; biscuits, bread; breakfast cereals; butter and margarine; cakes, muffins and pastry; canned fish and seafood; cereal bars; cheese; chewing gum; chilled fish; chocolate and sweets; coffee and tea; cooking oil; cordials; couscous; cream; crisps and snacks; desserts; eggs; electrolyte drinks; frozen fish, fruits; fruit and vegetable juices; honey and syrups; ice cream and edible ices; jam and spreads; jelly; maize (corn); mayonnaise/dressings; meal replacements; meat alternatives; milk; noodles; nuts and seeds; other; other salt; pasta; pizza; pre-prepared salad and sandwiches; processed meat and derivatives; ready males; rice; sauces; soft drinks; soups; spreads; unprocessed cereals; vegetables; waters; and yogurt products.

### 2.2. Identifying Free Sugars within the Ingredient Lists

Following the WHO definition of free sugars [23], ingredient lists of all products were systematically inspected for free sugar ingredients (FSI). All identified FSI and their varieties are listed in Supplementary Materials Table S1. Despite being used as food sweeteners, artificial sweeteners and sugar alcohols are neither monosaccharaides nor disaccharides and were therefore not considered as free sugars.

### 2.3. Assesment of Total and Free Sugar Content

Nowadays, nutrition declarations on pre-packaged products contain information on the (total) sugar content across the entire EU. However, labelling of the sugar content on pre-packaged foods sold across the EU market only became mandatory in 2017, despite the information having been voluntarily presented on most pre-packaged foods by then. The CLAS database was compiled using all available information from nutritional declarations on food labels. We also collected information provided in the lists of ingredients, which included all the ingredients in a certain product, in descending order of weight. By law, the indication of the quantity of an ingredient is required if the ingredient (a) appears in the name of the food; (b) is emphasized on the labelling in words, pictures or graphics; or (c) is essential to characterize a food and to distinguish it from products with which it might be confused because of its name or appearance. Considering that nutrition declarations do not provide information on the amount of free sugar content, such information can only be calculated or estimated based on the ingredient list and total sugar content of each product.

The free-sugar decision algorithm was established based on the previously published algorithm developed by Bernstein and colleagues for the Canadian food market analyses [38] (Table 1). In Steps 1 and 2, all products that contained 0 g of total sugar (e.g., cooking oils, eggs, plain rice) or contained no ingredients that would contribute to free sugar content were assigned 0 g of free sugar. In Step 3, all products that contained no ingredients with more than 0.5 g of naturally-occurring sugar per 100 g had all their total sugar assigned as free sugar. Among the remaining food products, those that had a complete ingredient list with percentages of FSI and/or ingredients with naturally-occurring sugars available, were assigned under Step 4. The equation used for calculating free sugar content is presented in Table 1. While Steps 1–4 were considered to be objective, the decisions for Steps 5–7 had to be made subjectively. Thus, the confidence of the calculated/estimated free sugar value decreased with each step. Products that could not be assigned to Steps 1–4, but had an unsweetened comparison available within the same category (e.g., a fruit yogurt compared with a plain yogurt), had a free sugar value estimated based on the naturally-occurring sugar content of the unsweetened product (Step 5). When the unsweetened comparison was non-existent, two options remained. If a comparable product that had already been assigned a free sugar value was available, a value reflective of the proportion of free sugar in the comparison product was assigned to the remaining product (Step 6). If an estimation of free sugar content was impossible from Steps 1–6, the free sugar values were estimated based on the order of ingredients on the ingredient lists (Step 7). All calculations in the paper were based on food composition data available from the Slovenian food composition database [43].

For certain food products such as condensed soups, the amount of total and free sugar was calculated for the “as consumed” form of the product to enable comparison with other food products within the same category. The calculations were based on the preparation instructions written on the product, while both the dilution factor and additional ingredients were considered.

**Figure 1:**
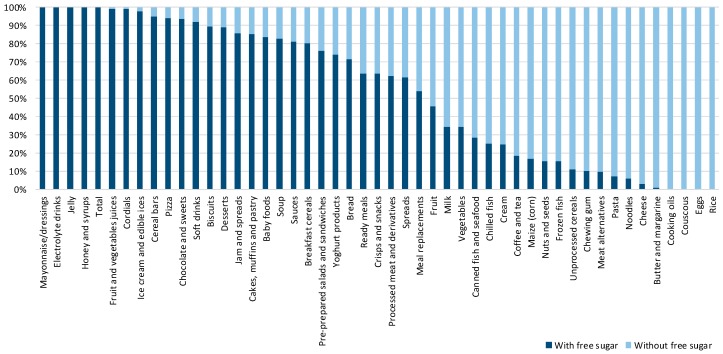
Proportion of packaged foods and beverages containing free sugar ingredients sorted by category.

**Figure 2:**
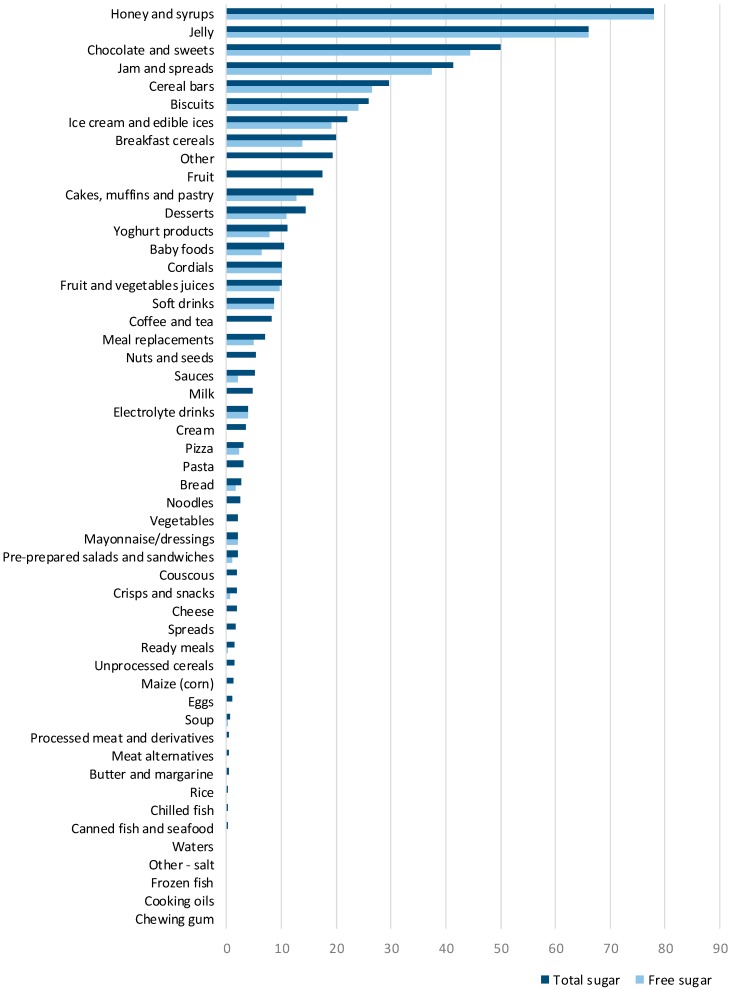
Median total and free sugar content (g/100 g or g/100 mL) per food category.

**Figure 3:**
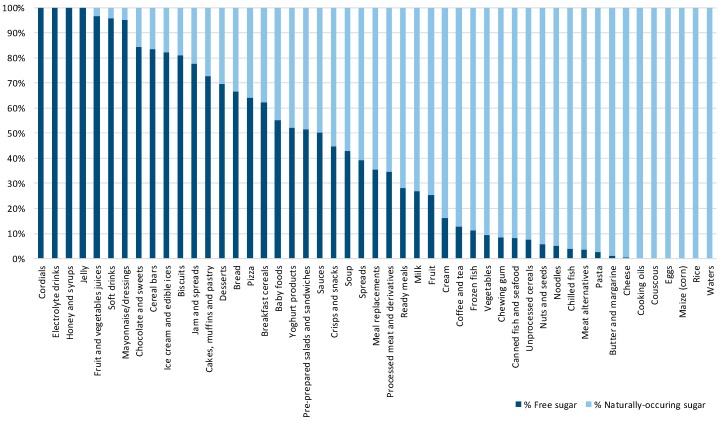
The amount of free sugar as a proportion of total sugar (%) per food category.

**Figure 4:**
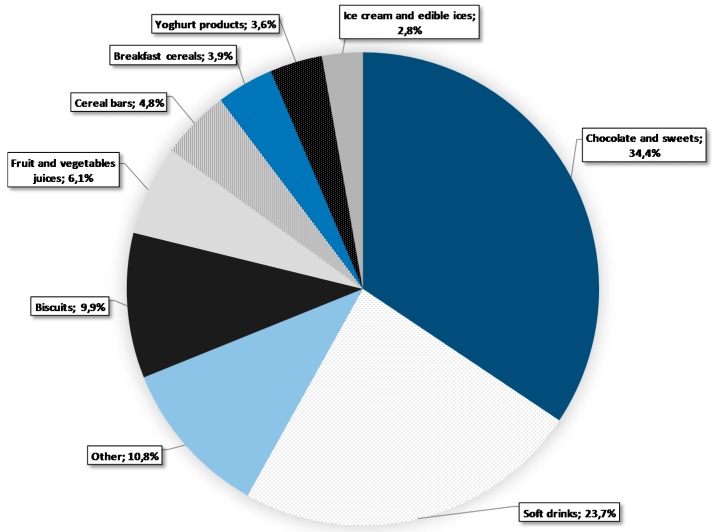
Relative contribution of different categories to the amount of free sugar sold.

While the proportions of products containing free-sugar ingredients were calculated with the full sample of eligible items (Figure 1, *N* = 10.674), estimations of the contents of free sugar were calculated only for products labelled with both an ingredient list and nutrition declaration (Table 1 and Table 2, Figure 2, Figure 3 and Figure 4; *N* = 9.405).

### 2.4. Share in Free Sugar Sales

**Figure 5:**
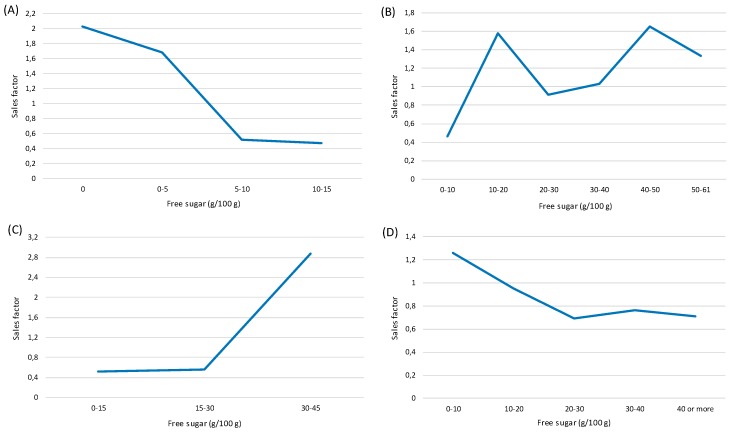

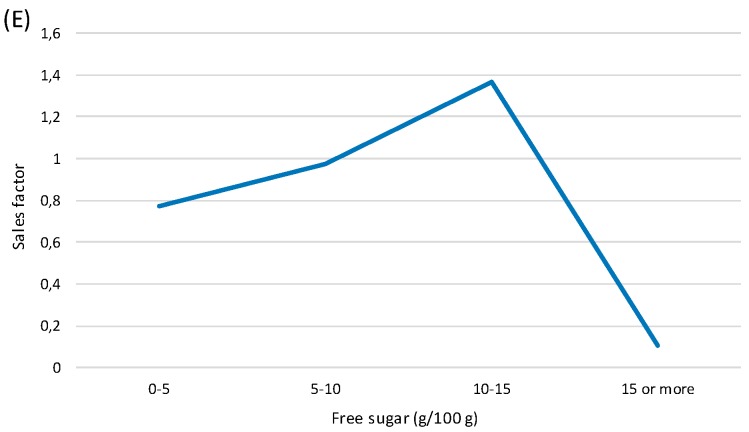
The correlation of the free sugar content and sales. (**A**) Yogurts; (**B**) Biscuits; (**C**) Breakfast cereals; (**D**) Cakes, muffins and pastry; and (**E**) Soft drinks.

The CLAS database was further complemented with country-wide 12 months sales data. Ensuring proper data handling, we obtained sales data from retailers covering the majority of the national market. The sales data referred to the national market and presented sales of food products for the 12 months period before the data collection (January 2014–December 2014). This was arranged on the condition that the results would not reveal sales data of any particular retailer. In this way we were able to access sales data for 8620 (91.7%) out of 10,674 products in the CLAS database, which were then included into analyses as reported in Figure 4 and Figure 5. The sales data were given in universal form, including EAN number, description of the product, the number of products sold per year, and the quantity of food (kg or L) per packaging. Matching foods between the databases was performed using EAN numbers. Using sales data from 2014 and the previously calculated free sugar content of each product, we evaluated the relative contribution of each food category. The final values representing shares in free sugar sales are presented as ratios between the total free sugar in all sold items in certain (sub) categories and the total free sugar in all sold items in the sample.

### 2.5. Sale-Weighted Total and Free Sugar Content

With the use of sales data acquired from the retailers and previously calculated free sugar content, we could analyze the correlation of total/free sugar content with sales data. Analyses were conducted using a sample of foods for which both the total/free sugar and sales data were available (*N* = 6563). Sales-weighted total/free sugar contents were calculated on the food category level (in g per 100 g or mL; results provided in the Supplementary Materials Table S2). Based on the priorities of the SRNPAH 2015–2025, five different food categories were chosen for in-depth analysis: (A) yogurts; (B) biscuits; (C) breakfast cereals; (D) cakes, muffins and pastry; and (E) soft drinks. All items within each selected food category were assigned to an appropriate group that represented a certain interval in the free sugar range. The intervals for each category were determined based on the free sugar range of all items within the category. Sales factors were calculated from the total number of products sold, and corrected by the number of items per interval. The final factor was given as the proportion of the average sales volume of the interval.

### 2.6. Statistical Analysis

The data were processed and evaluated using the computer programs Microsoft SQL Server Management Studio V13.0, Microsoft Analysis Services Client Tools 13.0, Microsoft Data Access Components (MDAC) 10.0, Microsoft Excel 2016 (Redmond, WA, USA), and the program tool CLAS V1.0 (Composition and Labelling Information System; Nutrition Institute, Ljubljana, Slovenia). For the purpose of statistical analyses, products’ nutritional values from the CLAS database were exported in the form of Microsoft Excel spreadsheets. Mean values, standard deviation (SD), and quartiles (min, 25th, 50th, 75th, max) were calculated for the total and free sugar content. Sales-weighted total/free sugar contents were given as an exact value and, therefore SDs are not provided. The proportion of free sugar-containing items was presented as a percentage (%) of all products within each category. The average amount of free sugar in an individual food category was expressed as a percentage (%) of the averaged total sugar content of the group. The impact of free sugar content on sales was analyzed using the analysis of variance.

## 3. Results

### 3.1. Proportion of Free Sugar-Containing Products in Different Food Categories

Out of 10,674 packaged foods and non-alcoholic beverages, a total of 10,563 met the inclusion criteria for the statistical analyses. In total, 52.6% of all analyzed products contained free sugar. The highest proportion of free sugar-containing items (i.e., 100%) was found among mayonnaise/dressing, electrolyte drinks, jellies, honey and syrups, and fruit and vegetables juices (Figure 1), however, it should be noted that some of these food categories cannot be manufactured without free sugar. Out of the rest, food categories with the highest proportion of pre-packed products containing free sugar were cordials (98.9%), ice cream and edible ices (97.8%), cereal bars (95.1%), and pizzas (94.1%). Figure 1 does not include categories where free sugar-containing products accounted for less than 10% of all products. Results from all the analyzed food categories are presented in the Supplementary Materials.

### 3.2. Median Total Sugar and Free Sugar Content of Packaged Foods and Non-Alcoholic Beverages

The median total and free sugar contents of different food categories with a median total sugar higher than 1.5 g/100 g are shown in Figure 2. Overall, the median free sugar was 0.3 g/100 g or /100 mL, while the median total sugar was 5.5 g/100 g or/100 mL (Table 2). When excluding products that did not contain total sugar, the median free sugar raised to 4.5 g/100 g or /100 mL, which was more than half of the median total sugar (7.5 g/ per 100 g/ or /100 mL) (data not shown). Honey and syrups, jellies, as well as chocolates and sweets had the highest median total sugar (78, 66, and 50 g/100 g, respectively) and median free sugar value (78, 66, and 44.4/100 g, respectively). For jam and spreads, cereal bars, biscuits, ice cream and edible ices, and breakfast cereals, median total sugar content ranged from 20.0–41.4 g/100 g/ or /100 mL and the median free sugar content ranged from 13.7–37.5 g/100 g or /100 mL. The most profound difference between the median total and free sugar content was found among fruits, coffee and tea, and nuts and seed, with the median total sugar of 17.5, 8.3, and 5.4 g/100 g or /100 mL, respectively, and the median free sugar content of 0 g/100 g or /100 mL. Interestingly, fruit and vegetable juices contained higher amounts of total and free sugar compared to soft drinks.

### 3.3. Contribution of Free Sugar to the Total Sugar Content

Considering only sugar-containing items, free sugars contributed an average of 57.5% to the total sugar content (Table 2). In cordials, electrolyte drinks, honey and syrups, and jellies, free sugar contributed 100% of the total sugar, while in fruit and vegetable juices, soft drinks, and mayonnaise/dressings, at least 95% of sugar came from free sugars (Figure 3). Additionally, free sugar accounted for more than 50% of the total sugar in the following food categories: chocolate and sweets (84.4%), cereal bars (83.4%), ice cream and edible ices (82.1%), biscuits (80.9%), jam and spreads (77.6%), cakes, muffins and pastry (72.8%), desserts (69.5%), bread (66.5%), pizzas (64.1%), breakfast cereals (62.3%), baby foods (55.1%), yogurt products (52.1%), pre-prepared salads and sandwiches (51.4%), and sauces (50.2%).

### 3.4. Share in Free Sugar Sales

Different food categories have different importance when it comes to the amount of free sugar consumed in our diets. With the use of sales data, we calculated the amount of free sugar (kg) sold per food category. The results, presented as relative values, are summarized in Figure 4. Chocolate and sweets and soft drinks alone accounted for 58.1% of all free sugar sold, followed by biscuits (9.9%), fruit and vegetable juices (6.1%), cereal bars (4.8%), breakfast cereals (3.9%), yogurt products (3.6%), and ice creams and edible ices (2.8%). All the remaining categories with a share in sales of less than 2.8% were combined under the category of other.

### 3.5. Correlation of the Free Sugar Content in Foods and Sales

Using yearly nation-wide sales data provided by the retailers, the correlation of free sugar content with sales was calculated, focusing on the following food categories: Yogurts, Biscuits, Breakfast cereals, Cakes, muffins and pastry, and Soft drinks (Figure 5). Additionally, the sales-weighted total/free sugar contents were calculated (in g per 100 g or mL; see the Supplementary Table S2 for data for all food categories). The results showed a very specific sales pattern with regard to the amount of free sugar for each food category. In the category of yogurts (Figure 5A), plain yogurt varieties were the preferred choice of consumers and the quantity of products sold in this category declined with the amount of free sugar content. This was also seen from the sales-weighted free sugar content (1.5 g/100 g), which was about 4-times higher than the mean free sugar level. A similar declining trend was also observed in a group containing cakes, muffins and pastry (Figure 5D), but it was much less pronounced when compared to yogurts. Sales-weighted free sugar content (14 g/100 g) was also lower than the mean free sugar level. A less favorable trend was recorded in categories containing biscuits (Figure 5B) and breakfast cereals (Figure 5C). The sales data for the latter showed that market-leading brands contained the highest amount of free sugar (30–45 g/100 g), while biscuits exhibited two separate picks in sales: in the intervals from 10–20 and 40–50 g/100 g, respectively. In both categories, sales-weighted free sugar contents were also notably higher (25.6 g and 26.9 g per 100 g of breakfast cereals and biscuits, respectively) than the mean free sugar levels. Among the soft drinks, consumers were least likely to buy products containing extremely high free sugar levels (>15 g of free sugar per 100 g), although products with very low free sugar (0–5 g/100 g) were also bought less frequently. Market-leading drinks contained approximately 10–15 g of free sugars. The sales-weighted free sugar level for this category was 8.4 g/100 mL, about 10% higher than the mean free sugar content (7.7 g/100 mL).

## 4. Discussion

In concordance with recommendations on free sugar consumption postulated by different national and international health organizations, researchers have attempted to estimate the added/free sugar content of pre-packaged foods and beverages in several countries [38,44,45,46]. The precise estimation of free sugar content in the available food supply is a necessary first step towards more informative studies focusing on per-capita free sugar consumption and follow-up strategies. This study was carried out as a starting point for future projects investigating free and total sugar intakes as well as potential sugar-content fluctuations in the food supply. While this cross-sectional study did not cover the entire national food market, a systematically inventoried subset of all foods and beverages found in the three largest grocery chains accounted for the majority of the Slovenian food supply of pre-packaged foods and non-alcoholic beverages. Our data showed that more than 50% of all 10,563 investigated products on the market contained one or more free sugar ingredients. This was somewhat lower when compared to the percentage of free sugar ingredients used in the US (74%) and Canada (65.4%) [38,47]. We identified 65 different types of free sugar ingredients listed on the ingredient lists, which, as already pointed out by Bernstein and colleagues [38], presents a great challenge for consumers trying to avoid excessive sugar consumption. Moreover, in an average sugar-containing product, 57.5% of all the sugar can be attributed to free sugar. The median free sugar content of products containing free sugar ingredients was 4.5 g per 100 g/100 mL.

Beyond the calculations of free sugar content, we also calculated the relative contribution of each food category to the total amount of free sugar sold. Results showed that chocolate, sweets and soft drinks accounted for nearly 60% of all free sugar sold in the three main grocery chains, possibly representing major culprits for excessive free sugar intake in Slovenia.

Using the sales data, we also investigated a possible correlation between free sugar content and the sales data provided by the retailers. Focusing on five food categories, we observed rather specific consumer preference for each food category regarding free sugar content. Brands of breakfast cereals with the highest amounts of free sugar were, for example, a preferred choice when it came to Slovenian consumers. However, it should be noted that this observation cannot be attributed to the effect of free sugar content alone and does not necessarily point to a causation, as the consumers’ purchasing decisions are driven by a variety of very different factors including brand, price, and packaging. While the exact effect of free sugar content on sales needs to be further investigated, the reported results in some food categories, such as breakfast cereals and biscuits, remain rather concerning.

The data obtained in this study will serve as a foundation for calculations of free sugar consumption as part of an ongoing national dietary intake survey. The outcomes of this survey will provide the first reliable data on free sugar consumption amongst different age groups and genders in Slovenia. These results will be needed when preparing further interventions aiming at lower sugar consumption.

A major strength of this study is the extent of the data collection and employment of yearly product-specific sales data, which have provided a very interesting insight into free sugar content in market-leading brands. The compiled database will be useful for various purposes and will support efficient monitoring of the changes in the food supply, and more reliable dietary assessments in the population. One major limitation of our study was the use of sugar content data as reported on food labels (rather than chemical analyses). However, there is no simple accurate analytical method that would enable measurements of free sugar content. Even measurements of total sugar would not be feasible on such a large scale. Therefore, we had to rely on the information provided by food manufacturers written on food labels, which is, however, a subject of regular inspection by the food authorities. In relation to the source of the data, another limitation of the study was that we were not able to estimate the content of free sugar in all of the products. Some foods with free sugar (based on information provided in the mandatory list of ingredients) were not labelled with nutritional declarations and were therefore excluded from our analyses. However, less than 12% of the foods (*N* = 1269) in the total sample were excluded due to missing data on sugar levels. It should be also noted, that results on sugar content are provided in grams per 100 g, and not per portion size, because portion size measures are not standardized in Slovenia, nor in European Union. Additionally, such a presentation allows for an easier comparison of the data collected in different countries.

## 5. Conclusions

In conclusion, our study was the first to show the extent of free sugar ingredients used in Slovenian pre-packaged foods and beverages. Using a step-by-step methodology, we were able to estimate the free sugar content for a large representative sample of items found on the market. The data obtained in the course of this study provided a valuable overview on the amount of sugar present in commonly consumed food and beverages, thus supporting interventions aimed at lowering free sugar consumption.

## Figures and Tables

**Table 1:** The following step-by-step method was used to estimate the free sugar content of packaged foods. The method was adapted from Bernstein and colleagues [38]. *n* (%) indicates the number and proportion of products calculated at each step.

Step	Description	*n* (%)
1	Products with 0 g of total sugar as declared on the nutrition facts label. Free sugar value = 0 g/100 g.	676 (7.2%)
2	Products that have no FSI listed on the ingredient list. Free sugar value = 0 g/100 g.	3759 (40%)
3	Products that contain no ingredients with naturally-occurring sugars. Free sugar value = 100 g/100 g.	1603 (17%)
4	Products with detailed lists of ingredients: % of FSI and/or % of ingredients with naturally occurring sugar available. The following formula was used to calculate free sugar content: Total sugar − Calculated naturally-occurring sugar = Free sugar	942 (10%)
5	Products that contain free sugar ingredients as well as naturally-occurring sugars and have an unsweetened alternative available in the same category (i.e., sweetened yogurt vs. plain yogurt). The following formula was used to calculate free sugar content: $\frac{100\times \left(\mathrm{sugar}\;\mathrm{per}\;100\;g\;\mathrm{unsweetened}-\mathrm{sugar}\;\mathrm{per}\;100\;g\;\mathrm{sweetened}\right)}{\left(\mathrm{sugar}/100\;g\;\mathrm{sweetened}-100\right)}$	873 (9.3%)
6	Products without unsweetened comparison but with a very similar product for which free sugar value had already been calculated were assigned a value reflective of the proportion of free sugar in the comparing product.	37 (0.4%)
7	Products that remained after all the other steps had been performed had their free sugar values estimated based on their lists of ingredients. The following formula was used to calculate free sugar content: Total sugar − Estimated naturally-occurring sugar = Free sugar	1385 (14.7%)

**Table 2:** Total and free sugar content (g/100 g or g/100 mL) of 10,407 packaged food products divided by food categories. Mean values, SD, quartiles (min, 25th, 50th/median, 75th, max), and mean free sugar as a proportion of free sugar are shown for each category.

	*n*	Total Sugar (g/100 g or g/100 mL)						Free Sugar (g/100 g or g/100 mL)						Free Sugar (% of Total Sugar)
Food Category		Mean (SD)	Min	25th	50th	75th	Max	Mean (SD)	Min	25th	50th	75th	Max	
Baby foods	104	13.3 (12.4)	0	8.1	10.4	11.8	53	7.2 (7.5)	0	3.7	6.4	8.7	43.3	56.2
Biscuits	736	24.6 (15.3)	0	9.5	26	36.6	60.8	22.4 (15.2)	0	5.5	24	35.2	60.8	81.2
Bread	161	3.1 (2.2)	0.2	1.3	2.7	4	14	2.2 (2.3)	0	0	1.6	3.3	14	66.5
Breakfast cereals	297	19.2 (12.1)	0.3	9	20	27.6	60	14.7 (13.1)	0	1.9	13.7	23.8	57.4	62.3
Butter and margarine	95	0.5 (0.8)	0	0	0.5	0.5	5.1	0 (0.1)	0	0	0	0	0.5	1.5
Cakes. muffins and pastry	360	18.7 (15.1)	0.2	3.6	15.8	32.8	60.7	16.5 (15.1)	0	1.8	12.8	29.5	60.3	72.8
Canned fish and seafood	165	0.9 (1.4)	0	0	0.1	1.1	7.4	0.3 (0.9)	0	0	0	0	5.4	12.6
Cereal bars	61	28.4 (12.2)	5.1	22.7	29.6	34.1	69.9	23.8 (12.6)	0	15.5	26.5	30.4	67.7	83.4
Cheese	459	2.1 (2.6)	0	0.1	1.8	3.5	36	0.1 (1.5)	0	0	0	0	32	0.5
Chewing gum	59	6.5 (20.9)	0	0	0	0	86	6.3 (20.9)	0	0	0	0	86	71.4
Chilled fish	44	0.4 (0.8)	0	0	0.1	0.5	3.1	0.1 (0.5)	0	0	0	0	3.1	5.2
Chocolate and sweets	1078	49.3 (19.2)	0	42.7	50	58.2	99.9	44.6 (20.9)	0	36.2	44.4	55.5	99.9	87.1
Coffee and tea	560	23.5 (27.3)	0	0.2	8.3	52	92	5.5 (16)	0	0	0	0	92	12.9
Cooking oils	126	0 (0)	0	0	0	0	0	0 (0)	0	0	0	0	0	0
Cordials	92	31.9 (32.4)	0	9.2	10.1	73.2	90	31.9 (32.4)	0	9.2	10.1	73.2	90	100
Couscous	12	1.8 (0.7)	0.8	1	1.9	2.3	2.7	0 (0)	0	0	0	0	0	0
Cream	106	4.3 (2.9)	0	3	3.4	4.2	15	1.5 (3.5)	0	0	0	0	15	16.4
Crisps and snacks	242	3 (4.9)	0	1	1.8	3.4	52.3	2.1 (4.7)	0	0	0.7	2.8	50.6	45.6
Desserts	182	16.2 (8.8)	0	13	14.4	16.3	55	12.1 (9.5)	0	8.7	10.9	13.6	55	70
Eggs	22	0.8 (0.3)	0	0.9	1	1	1	0 (0)	0	0	0	0	0	0
Electrolyte drinks	12	9.9 (18.9)	3.9	4	4	5.1	70	9.9 (18.9)	3.9	4	4	5.1	70	100
Frozen fish	78	0.5 (0.8)	0	0	0	0.5	2.9	0.2 (0.6)	0	0	0	0	2.9	18.7
Fruits	248	32.7 (24)	0.1	12.3	17.5	57.2	78	8.2 (16.5)	0	0	0	9	76	25.4
Fruit and vegetables juices	264	9.5 (2.9)	0.1	8.4	10	11.2	17	9.1 (3.1)	0	8	9.7	11.1	17	96.8
Honey and syrups	12	78 (3.2)	75	75	78	80.9	80.9	78 (3.2)	75	75	78	80.9	80.9	100
Ice cream and edible ices	226	21.4 (6.1)	3.4	19.7	22	24.8	41	18.1 (6.1)	0	15.9	19.2	21.7	37.2	82.1
Jam and spreads	182	41.2 (15.7)	2.5	35.5	41.4	54	53	35.9 (17.7)	0	31.9	37.5	49.4	60.6	77.6
Jelly	6	62.9 (7.4)	53	56.9	66	67.8	70	62.9 (7.4)	53	56.9	66	67.8	70	100
Maize (corn)	6	1.1 (0.7)	0.1	0.8	1.3	1.6	1.7	0 (0)	0	0	0	0	0	0
Mayonnaise/dressings	55	3 (2.9)	0.1	1.5	2.1	3	12.2	2.7 (2.5)	0.1	1.5	2	3	12.2	95.1
Meal replacements	13	21.5 (22)	4	5	6.9	52.2	52.8	11.1 (14)	0	0	5	30.5	30.8	35.4
Meat alternatives	61	0.9 (1.5)	0	0.2	0.5	1	9.1	0.1 (0.4)	0	0	0	0	2.4	5.4
Milk	181	6.2 (8.8)	0.1	4.2	4.8	6.4	93.2	2.5 (8.3)	0	0	0	2.8	93.2	26.9
Noodles	116	2.2 (1.4)	0	1.1	2.5	3.5	6.2	0 (0.2)	0	0	0	0	1.1	5.7
Nuts and seeds	174	11 (13.7)	0	3.9	5.4	7.1	51	2.4 (9.1)	0	0	0	0	51.2	5.8
Pasta	326	2.8 (1.5)	0.2	1.7	3.1	3.6	10	0.1 (0.5)	0	0	0	0	4.4	2.6
Pizza	33	3.2 (1.3)	0.3	2.15	3.1	4.1	5.6	2.2 (1.3)	0	1.4	2.2	3.1	4.6	64.1
Pre-prepared salads and sandwiches	46	2.2 (1)	0.7	1,4	2	2.7	4.7	1.2 (1.2)	0	0	1.1	2	3.5	51.4
Processed meat and derivatives	772	0.4 (0.4)	0	0.1	0.5	0.5	3.8	0.2 (0.4)	0	0	0	0.4	3.7	39.3
Ready meals	292	2.2 (3.1)	0	0.8	1.5	2.6	38	0.8 (1.9)	0	0	0.1	0.8	17.7	28.2
Rice	29	0.4 (0.4)	0	0.2	0.3	0.5	2.4	0 (0)	0	0	0	0	0	0
Sauces	310	9.9 (12.1)	0	2.8	5.2	9.4	60.9	6.8 (11.5)	0	0	2.1	5.9	60.7	52.1
Soft drinks	381	7.7 (3.8)	0	5.6	8.6	10	30	7.7 (3.8)	0	5.6	8.6	10	30	99.9
Soup	171	0.7 (0.7)	0	0.2	0.6	0.8	4.3	0.4 (0.6)	0	0	0.3	0.5	3.9	42.8
Spreads	276	13.4 (21.2)	0	0.6	1.6	9.5	64.3	8.7 (17.9)	0	0	0	2.7	58.5	39.3
Unprocessed cereals	110	2.12 (2.18)	0.2	0.9	1.46	2.4	15	0.3 (1)	0	0	0	0	4	7.5
Vegetables	530	3 (4.1)	0	0.25	2.1	3.9	26.5	0.54 (1.6)	0	0	0	0	13.7	9.7
Waters	82	0 (0)	0	0	0	0	0	0 (0)	0	0	0	0	0	0
Yogurt products	449	10.2 (4.3)	0	5.3	11.1	13.1	20.8	6.5 (4.7)	0	0	7.9	10.1	17.1	52.3
Total	10,407	15.1 (19.7)	0	1.2	5.5	22	99.9	10.3 (17.7)	0	0	0.3	11	99.9	57.5

## Supplementary Materials

The following are available online at http://www.mdpi.com/2072-6643/10/2/151/s1. Supplementary Table S1: The list of all free sugar ingredients (FSI) identified during the analyses of pre-packaged products in the CLAS (Composition and Labelling Information System) database. Supplementary Table S2: Mean total sugar and Mean free sugar content (in g per 100 g or mL) of packaged food products divided by food categories.
