# Supplementary material for: Total and Free Sugar Content of Pre-Packaged Foods and Non-Alcoholic Beverages in Slovenia
Source: Nutrients. 2018 Jan 30;10(2):151. doi: 10.3390/nu10020151 (PMC5852727; doi:10.3390/nu10020151)
Supplement: Supplementary file 1 [file nutrients-10-00151-s001.pdf]

Zupanič et al. Total and Free Sugar Content of Pre-Packaged Foods and Non-Alcoholic Beverages in Slovenia. Nutrients 2018, 10(2), 151; http://dx.doi.org/10.3390/nu10020151

Supplementary Table 1. The list of all free sugar ingredients (FSI) identified during the analyses of pre-packaged products in the CLAS (Composition and Labelling Information System) database.

Free sugar ingredients (FSI)	All varieties of FSI
Sugar (sucrose) and sugar syrups	Sugar, sugar syrup, sucrose, cane sugar, raw cane sugar, cane sugar syrup, refined sugar, unrefined cane sugar, brown sugar, brown cane sugar, inverted sugar, inverted sugar syrup, caramelized sugar, caramelized sugar syrup, powdered sugar, crystalized sugar, beetroot sugar
Glucose	Glucose, grape sugar, dextrose, glucose syrup, glucose rice syrup, glucose fruit syrup, glucose crystals
Fructose	Fructose, fructose syrup, dextrose, corn dextrose, wheat dextrose, oligofructose
Corn syrup	Corn syrup, dried corn syrup, high fructose corn syrup, fructose-glucose syrup
Honey	Honey, powdered honey, forest honey, flower honey, acacia honey, linden honey, bee honey
Fruit juice*	Fruit juice, concentrated fruit juice, fruit juice from concentrate, fruit juice concentrate, fruit purée
Agave	Agave syrup, agave concentrate
Molasses	Molasses, cane sugar molasses
Other syrups and malts	Barley syrup, barley malt, barley malt extract, wheat syrup, wheat glucose syrup, wheat malt, wheat malt extract, wheat malt flour, rice syrup, corn malt, maple syrup
Other sugars	Maltose, palm sugar, coconut sugar, added lactose

***Notes:** Includes fruit juices (e.g., orange juice, apple juice, pineapple juice). [1] Fruit purées had 50% of total sugar assigned as free sugar.

Supplementary Table 2: Mean total and Mean free sugar content (in g per 100 g or mL) of packaged food products divided by food categories (Slovenia, 2015)

	Full sample		Sample with available sale data			
	Mean sugar content		Mean sugar content		Sale weighted sugar content	
Food category	Mean Total sugar	Mean Free sugar	Mean Total sugar	Mean Free Sugar	Total Sugar	Free Sugar
Baby foods	13,3	7,2	13,3	7,3	12,9	7,9
Biscuits	24,6	22,4	23,7	22,0	28,1	26,9
Bread	3,1	2,2	3,4	2,7	3,4	2,3
Breakfast cereals	19,2	14,7	19,0	15,1	28,2	25,6
Butter and margarine	0,5	0	0,5	0,0	0,6	0
Cakes, muffins and pastry	18,7	16,5	18,9	17,6	15,4	14
Canned fish and seafood	0,9	0,3	0,8	0,3	1	0,5
Cereal bars	28,4	23,8	28,9	24,5	28,4	25,9
Cheese	2,1	0,1	2,3	0,2	2,5	0
Chewing gum	6,5	6,3	8,1	7,9	5,1	5
Chilled fish	0,4	0,1	0,5	0,2	0,6	0,3
Chocolate and sweets	49,3	44,6	48,7	44,8	59,8	57,8
Coffee and tea	23,5	5,5	29,1	24,0	34,8	29,5
Cooking oils	0	0	0,0	0,0	0	0
Cordials	31,9	31,9	29,2	29,2	13,5	13,5
Couscous	1,8	0	1,7	0,0	1,9	0
Cream	4,3	1,5	4,4	1,9	3,8	0,6
Crisps and snacks	3	2,1	3,1	2,2	2,5	0,9
Desserts	16,2	12,1	16,2	13,5	14,8	11,8
Eggs	0,8	0	0,8	0,0	0,8	0
Electrolyte drinks	9,9	9,9	9,9	9,9	4,7	4,7
Frozen fish	0,5	0,2	0,6	0,3	0,7	0,2
Fruit	32,7	8,2	34,0	10,7	31,1	6,9

	Full sample		Sample with available sale data			
	Mean sugar content		Mean sugar content		Sale weighted sugar content	
Fruit and vegetables juices	9,5	9,1	9,4	9,0	9,8	9,4
Honey and syrups	78	78	/	/	/	/
Ice cream and edible ices	21,4	18,1	22,3	19,0	22,5	19,3
Jam and spreads	41,2	35,9	40,2	34,7	40,7	37,1
Jelly	62,9	62,9	66,7	66,7	65,3	65,3
Maize (corn)	1,1	0	0,1	0,0	0	0
Mayonnaise/dressings	3	2,7	3,1	2,8	1,7	1,6
Meal replacements	21,5	11,1	7,6	2,5	4,5	0,6
Meat alternatives	0,9	0,1	0,9	0,1	0,5	0
Milk	6,2	2,5	5,7	2,2	4,7	0,1
Noodles	2,2	0	2,3	0,1	1,4	0
Nuts and seeds	11	2,4	9,3	1,8	5,6	0,7
Other	32,2	20,1	38,7	37,5	40,8	40,7
Other—salt	0,4	0,4	0,5	0,4	0	0
Pasta	2,8	0,1	2,8	0,1	3,2	0
Pizza	3,2	2,2	3,0	2,0	3,7	2,6
Pre-prepared salads and sandwiches	2,2	1,2	2,2	2,0	1,8	2
Processed meat and derivatives	0,4	0,2	0,5	0,4	0,3	0,2
Ready meals	2,2	0,8	2,2	0,8	1,5	0,4
Rice	0,4	0	0,4	0,0	0,4	0
Sauces	9,9	6,8	9,3	7,7	7,6	5,9
Soft drinks	7,7	7,7	7,9	7,8	8,4	8,4
Soup	0,7	0,4	0,3	0,2	0,5	0,3
Spreads	13,4	8,7	14,2	12,3	39,5	36,7
Unprocessed cereals	2,1	0,3	2,2	0,5	1,7	0,7
Vegetables	3	0,54	2,7	0,7	2	0,5
Waters	0	0	0,0	0,0	0	0
Yoghurt products	10,2	6,5	9,8	6,2	5,8	1,5
of which fruit yoghurts	12,2	8,88	11,9	8,6	12,3	8,4
Total	15,1	10,3	11,2	9,0	11,4	9,4
